# Methodological aspects of MRI of transplanted superparamagnetic iron oxide-labeled mesenchymal stem cells in live rat brain

**DOI:** 10.1371/journal.pone.0186717

**Published:** 2017-10-19

**Authors:** Daria Namestnikova, Ilya Gubskiy, Irina Kholodenko, Pavel Melnikov, Kirill Sukhinich, Anna Gabashvili, Daniil Vishnevskiy, Anastasia Soloveva, Maxim Abakumov, Igor Vakhrushev, Alexei Lupatov, Vladimir Chekhonin, Leonid Gubsky, Konstantin Yarygin

**Affiliations:** 1 Pirogov Russian National Research Medical University, Moscow, Russia; 2 Institute of Biomedical Chemistry, Moscow, Russia; 3 Serbsky Federal Medical Research Centre of Psychiatry and Narcology, Moscow, Russia; 4 Koltzov Institute of Developmental Biology, Moscow, Russia; 5 National University of Science and Technology, Moscow, Russia; Fraunhofer Research Institution of Marine Biotechnology, GERMANY

## Abstract

*In vivo* tracking of transplanted mesenchymal stem cells (MSCs) migration and homing is vital for understanding the mechanisms of beneficial effects of MSCs transplantation in animal models of diseases and in clinical trials. Transplanted cells can be labeled with superparamagnetic iron oxide (SPIO) particles and visualized in vivo using a number of iron sensitive MRI techniques. However, the applicability of those techniques for SPIO-labeled MSCs tracking in live brain has not been sufficiently investigated. The goal of this study was to estimate the efficiency of various MRI techniques of SPIO-labeled cell tracing in the brain. To achieve that goal, the precision and specificity of T2WI, T2*WI and SWI (Susceptibility-Weighted Imaging) techniques of SPIO-labeled MSCs tracing *in vitro* and in live rat brain were for the first time compared in the same experiment. We have shown that SWI presents the most sensitive pulse sequence for SPIO-labeled MSCs MR visualization. After intracerebral administration due to limitations caused by local micro-hemorrhages the visualization threshold was 10^2^ cells, while after intra-arterial transplantation SWI permitted detection of several cells or even single cells. There is just one publication claiming detection of individual SPIO-labeled MSCs in live brain, while the other state much lower sensitivity, describe detection of different cell types or high resolution tracing of MSCs in other tissues. This study confirms the possibility of single cell tracing in live brain and outlines the necessary conditions. SWI is a method convenient for the detection of single SPIO labeled MSCs and small groups of SPIO labeled MSCs in brain tissue and can be appropriate for monitoring migration and homing of transplanted cells in basic and translational neuroscience.

## Introduction

Transplantation of cells (cell therapy) is an innovative biomedical technology which, together with tissue engineering, constitutes the core of the emerging field of regenerative medicine. Transplantation of autologous or allogeneic mesenchymal stem cells (MSCs) can induce symptom alleviation in animal models of many human neurological disorders and these results are in line with the outcomes of first clinical trials involving patients with certain central nervous system diseases, including ischemic stroke, multiple sclerosis, motor neuron disease, brain injury, and others [1–4]. Though transplantation of MSCs is effective, the cellular and molecular mechanisms of its beneficial effects have not been fully elucidated. Further research in this field, including development of reliable and accurate methods of the *in vivo* tracking of transplanted cells is needed.

Several imaging modalities for visualization of transplanted cells in live animals, including magnetic resonance imaging (MRI), positron emission tomography, single-photon emission computed tomography, and others, have been proposed and tested [5]. Among them, MRI combined with the labeling of cells with superparamagnetic iron oxide (SPIO) micro- or nanoparticles looks as one of the most expedient. This approach was first introduced more than 20 years ago [6,7] and since then further research aimed to improve this technique is ongoing [5,6]. Importantly, coated SPIO micro- and nanoparticles display low toxicity and have been successfully used as cell labeling agents in several MRI cell tracking studies in humans [8–11]. Due to broad availability of clinical magnetic resonance scanners, MRI of SPIO-labeled stem cells has a potential to evolve into a commonly accepted method of transplanted cells tracking in clinical trials of cell-based therapies and in other medical applications.

SPIO-labeled cells can be visualized with different MRI techniques including T2 weighted imaging (T2WI) [12], T2* weighted imaging (T2*WI) [13–15], and susceptibility-weighted imaging (SWI) (for review see [16,17]). SWI combines the information about local alterations of the phase and magnitude of the magnetic field [18]. Compared to techniques utilizing sequences based on just gradient echo, this method is more receptive to magnetic field inhomogeneity induced by the presence of paramagnetics and, therefore, has the potential to provide optimal traceability of SPIO-containing cells. Indeed, high sensitivity of SWI in SPIO-labeled cell tracking in cell transplantation experiments has been actually demonstrated [19–23].

A number of studies have shown the ability of MRI to detect single SPIO-labeled cells in phantom experiments [14,24–26] or *in vivo* after transplantation of cells into animals [13,27–30]. However, only the latter publication [13] describes detection of individual SPIO-labeled MSCs in live brain, while the other represent data regarding different cell types and/or tissues. Therefore, more research aimed to validate and further develop the MR method of SPIO-labeled MSCs detection in brain is needed in order to determine its detection limits and accuracy.

The goal of this study was to evaluate the applicability and precision of several MRI techniques, including T2WI, T2*WI based on MEDIC (Multi-Echo Data Image Combination) or FLASH 3D (Fast Low Angle Shot) pulse sequences, and SWI in SPIO-labeled MSC tracking in the phantom experiments *in vitro* and in live rat brain. We used cultured human mesenchymal stem cells (hMSCs) isolated from placenta, loaded with SPIO microspheres. Labeled cells were injected into phantoms made of plastic syringes filled with a collagen gel, delivered stereotaxically into the rat striatum or infused via the internal carotid artery. Labeled cells were visualized in the live brain or phantom using MRI techniques listed above. MR images captured *in vivo* were verified by histological methods. Imaging of different quantities of labeled hMSCs injected into the phantoms or brain tissue provided data concerning comparative sensitivity of different MRI techniques. MRI images obtained after intra-arterial cell administration provided the assessment of the suitability of MRI for the *in vivo* tracking of small groups of cells and individual cells in brain tissue.

## Materials and methods

### Cell isolation and culture

hMSCs were isolated from normal human placenta (38–40 weeks of gestation) obtained from informed consent mothers at the Research Center for Obstetrics, Gynecology and Perinatology (Moscow, Russian Federation) using a conventional procedure described elsewhere [31]. The study was approved by the local Ethical Committee of the Research Center for Obstetrics, Gynecology and Perinatology and the Pirogov Russian National Research Medical University. In brief, human placenta tissue samples were thoroughly minced with scissors, extensively washed with cold Hank’s solution and after gentle mechanical agitation incubated for 30 min at 37°C in Hank’s solution in the presence of 0.1% type I collagenase (Gibco). The suspension was then centrifuged at 300×g for 10 min at 4°C, supernatant was discarded and pellet resuspended in the complete culture medium comprising DMEM-F12, 2 mM L-glutamine, 100 U/ml penicillin, 0.1 mg/ml streptomycin and 10% fetal bovine serum (all reagents from Gibco). Cells were plated in T75 culture flasks and cultured in humidified atmosphere under standard conditions (37°C, 5% CO_2_). Cells were allowed to adhere for 3 days and non-adherent cells were removed by replacing the medium. Upon reaching 80–90% confluence, adherent hMSCs were harvested by trypsinization and subcultured at 1:3 ratio in T75 flasks.

### Cell labeling

hMSCs were labeled with SPIO-containing microspheres (MC03F Bangs Laboratories, mean diameter 0.50–0.99 μm) carrying Dragon Green fluorescent dye (λex = 480 nm, λem = 520 nm). Cell culture grown to 80–90% confluence was maintained for 12 hours in a CO_2_ incubator in the presence of SPIO microspheres (5 μl of the stock suspension supplied by the manufacturer per 1 ml of culture medium), washed twice with Hank’s solution to remove microspheres that had not been absorbed by the cells, harvested by trypsinization and used in the phantom and animal transplantation experiments. In the preliminary experiments different times of incubation of cells with MC03F microspheres and concentrations of microspheres were tested. If 5–10 μl of the stock suspension per 1 ml of the culture medium was added, labeling reached its maximum after 10 hours of incubation and remained unchanged for at least 36 hours. To verify cell labeling, fluorescent and transmitted light microscopy was performed the next day after procedure using an AxioPlan-2 (Zeiss) fluorescent microscope and Zeiss Axiovert 40CFL microscope. Prior to microscopic examination, cells were washed three times with PBS, fixed with 4% paraformaldehyde, and stained with DAPI to allow cell nuclei visualization with fluorescent microscopy. The efficacy of the uptake of MC03F microspheres was also evaluated by flow cytometry. hMSC culture grown to 80–90% confluence was labeled with MC03F microspheres as described above. Cells maintained without SPIO microspheres served as negative control. After incubation, the cells were washed twice with PBS to remove microspheres that had not been absorbed by the cell. Cells were then harvested with 0.25% trypsin-EDTA, centrifuged (1000 rpm for 5 min at 25°C) and resuspended in serum free DMEM F12 (Gibco) medium to a final cell concentration of 4x10^5^ cells/ml. The fluorescence intensity and light scattering of the Dragon Green fluorescent dye were measured by FACS-Aria flow cytometer/cell sorter (BD Bioscience).

In some experiments hMSCs labeled with SPIO microparticles were additionally labeled with PKH26 red fluorescent dye (Sigma-Aldrich) according to the manufacturer's protocol. Briefly, after labeling with SPIO microspheres cells were harvested and placed in a conical polypropylene tube at the concentration of 2x10^6^ cells per ml. Cells were washed with the serum-free medium, centrifuged for 5 minutes, and the supernatant was aspirated. hMSCs were resuspended in 1 ml of Diluent C. 1 ml of Diluent C was separately mixed with 4 μl of PKH26 and added to the hMSCs suspension. After incubation for 5 minutes, the labeling reaction was stopped by adding an equal volume of fetal bovine serum. Double-labeled cells were immediately transplanted into the animals.

### Evaluation of the viability and proliferation rate of SPIO labeled cells

Cells were seeded in 24-well cell culture plates (30,000 cells per well), left overnight to attach to plastic, and labeled with fluorescent magnetic microspheres as described above. Labeled hMSCs were rinsed three times in DMEM/F12 medium to remove the unbound label, fresh DMEM/F12 added, and cells were cultured for seven days under standard conditions, with medium exchange every two days. The number of viable cells was assessed with CytoTox 96 assay kit (Promega, USA; the assay is based on the measurement of the activity of lactate dehydrogenase (LDH) in live cells) immediately after completion of the labeling procedure (day 0) and after 1, 3, 5 and 7 days of culture, following the slightly modified manufacturer’s protocol. Briefly, the wells with labeled cells were washed three times with PBS and filled with 300 μl of deionized water. Cell membranes were lysed by three rounds of freezing and thawing. 50 μl aliquots of cell lysate were transferred into a 96-well plate, mixed with equal volume of substrate reagent supplied in the kit, and incubated for 1h at room temperature in the dark. The reaction was quenched by the addition of 50 μl of stop reagent (supplied in the kit). Optical density was measured at 490 nm using TECAN MX300 Pro plate reader. Cells subjected to exactly the same procedures, except that they were kept in the culture medium without microspheres, served as controls.

### Measurement of the iron content in SPIO-labeled cells

To determine the average content of iron per one SPIO-labeled cell, 5x10^5^ labeled hMSCs were pelleted by centrifugation and dissolved by incubation in 0.5 ml of the iron release reagent (0.5 M HCl, 2.25% w/v KMnO_4_) for 2 hours at 60°C. 250 μl of the resulting solution were mixed with 125 μl of H_2_O and 25 μl of the iron detection reagent (6.5 mM of the sodium salt of 3-(2-Pyridyl)-5,6-diphenyl-1,2,4-triazine-4′,4′′-disulfonic acid, 6.5 mM neocuproine, 2.5 М ammonium acetate, 1 M ascorbic acid). Light absorbance at 490 nm was measured with Victor X3 spectrophotometer (PerkinElmer) and iron concentration determined using the calibration curve created utilizing standard solutions with known iron content and expressed in pg iron per cell. Three independent experiments with separate cell preparations were carried out and results were presented as means of 3 experiments ± standard deviation.

### Cell transplantation into the rat brain

All manipulations with experimental animals were approved by the local Ethical Committee of the Pirogov Russian National Research Medical University (Protocol No 140 from December 15, 2014) and carried out in accordance with the directive 2010/63/EU on the protection of animals used for scientific purposes of the European Parliament and the Council of the European Union of September 22, 2010. Adult male Wistar rats weighing 250–300 g were obtained from AlCondi, Ltd animal breeding house, Moscow, Russian Federation. The animals (n = 39) were randomly attributed to one of the following seven experimental groups: transplantation of 10^1^ SPIO-labeled hMSCs (n = 5); transplantation of 10^2^ SPIO-labeled hMSCs (n = 8); transplantation of 10^3^ SPIO-labeled hMSCs (n = 4); transplantation of 10^4^ SPIO-labeled hMSCs (n = 7); transplantation of 10^5^ SPIO-labeled hMSCs (n = 8); saline injection (negative control, n = 4); transplantation of 10^5^ hMSCs double-labeled with SPIO and PKH26 (n = 3). For the surgery, rats were anesthetized with 3% isoflurane and maintained at artificial ventilation with the mixture of 2–2.5% isoflurane and 97.5–98% atmospheric air supplied by E-Z-7000 animal anesthesia system (E-Z Anesthesia® Systems). Throughout all surgical procedures, body temperature was maintained at 37°C with a heating pad. For cell transplantation, anesthetized rats were placed in the Angle Two computer-assisted stereotaxic system (Leica). After skin incision and removing of surface tissues, a hole was drilled in the skull and 20 μl of the suspension of SPIO-labeled hMSCs in saline or 20 μl of saline were slowly injected over the 5 min time interval using a 500 μl Hamilton microsyringe and an automatic syringe pump system (KD Scientific). Injection was made into the right striatum according to the following stereotactic coordinates: bregma +0.6 mm AP (anterior-posterior), +3.5 mm ML (medial-lateral), -4.5 mm VD (ventral-dorsal). After transplantation, the needle was left in place for 5 min to allow the cell suspension to distribute in the brain tissue and to minimize the reverse flow of transplanted material into the needle. The needle was slowly withdrawn and skin was closed with simple interrupted stitches.

#### Intra-arterial cell transplantation

Two groups of rats were administered with SPIO-labeled hMSCs (n = 5) or hMSCs double-labeled with SPIO microparticles and PKH26 (n = 3) intra-arterially. Surgery and cell transplantation were performed under isoflurane anesthesia as described in the previous section. The right common carotid artery (CCA), external carotid artery (ECA), and internal carotid artery (ICA) were exposed. Under the operation microscope microsurgical clips were placed on CCA and ICA, while the pterygopalatine artery (PPA) was ligated by a 5–0 silk suture. The ECA was then cut with micro-scissors and a polyurethane micro-catheter (Doccol corporation, outer diameter 0.635 mm, inner diameter 0.305 mm) filled with saline to prevent air bubbles was inserted into the stump of the ECA and after removal of the microsurgical clip advanced into the extracranial branch of ICA. The micro-catheter was fastened to ECA by a 5–0 silk suture. The microsurgical clip from CCA was removed to maintain the blood flow in the ICA. The catheter was then connected to a 1 ml syringe placed in the microinjector, and 5x10^5^ SPIO-labeled hMSCs suspended in 1 ml of PBS were delivered into the ICA over a 10-minute interval. After cell transplantation the catheter was removed, the stump was electrocoagulated, and incision was closed with a 3–0 silk suture. The described cell infusion parameters (cell dose not more that 5x10^5^ in 1 ml, infusion velocity 100 μl/1 min with maintenance of blood flow in ICA and ligation of PPA) were selected during a preliminary study [32] and proved to prevent serious adverse events such as cerebral embolism.

### Phantom for the *in vitro* MRI studies

*In vitro* MRI was carried out using phantoms made of 5 ml plastic syringes filled with Sphero®GEL (Biomir Service, Krasnoznamensk, Moscow region, Russia) and injected with 10^1^ hMSCs in 20 μl saline utilizing the stereotaxic system described in the previous section. Sphero®GEL is a gel material made of processed bovine collagen and certified for medical use. We did not try to inject single cells, because we considered it impossible to reproducibly deliver a single cell into a phantom.

### Magnetic resonance imaging (MRI)

MRI of rat brain for all experimental groups was performed immediately after cell transplantation with the 7T ClinScan system (Bruker BioSpin, USA), 30/12 cm bore size, 630 mT/m gradient strength, 6300 T/m/s maximum slew rate, with phased array four elements rat brain coil. During the procedure rats were maintained at inhalation anesthesia with the mixture of 2–2.5% isoflurane and 97.5–98% atmospheric air supplied by an EZ-7000 animal anesthesia system (E-Z Anesthesia® Systems). The following MRI protocols were used to visualize SPIO-labeled cells in the rat brain (the total duration was approximately 20 min.):

- T2 weighted imaging (T2WI; Turbo Spin Echo pulse sequence with restore magnetization pulse; turbo factor = 9; TR/TE = 4000/46 ms; averages = 2; spectral fat saturation; FOV = 37 x 29.6 mm; slice thickness = 0.5 mm; matrix size = 320 x 256; acquisition time = 4:44);
- T2* weighted imaging based on MEDIC pulse sequence (T2*WI based on MEDIC; MEDIC—Multi Echo Data Image Combination with RF spoiling pulse sequence; combined echoes = 5; TR/TE = 1540/30 ms; flip angle = 30; averages = 1; spectral fat saturation; FOV = 37x29.6 mm; slice thickness = 0.5 mm; matrix size = 320 x 256; acquisition time = 6:34);
- T2* weighted imaging based on FLASH 3D pulse sequence (T2*WI based on FLASH 3D; 3D Gradient Echo pulse sequence with RF spoiling and flow compensation; TR/TE = 50/19.1 ms; flip angle = 15; averages = 1; spectral fat saturation; FOV = 30 x 20.6 mm; slice thickness = 0.5 mm; matrix size = 256 x 176; acquisition time = 5:31);
- Susceptibility Weighted Imaging (SWI; 3D Gradient Echo with RF spoiling and flow compensation; TR/TE = 50/19.1 ms; flip angle = 15; averages = 1; spectral fat saturation; FOV = 30 x 20.6 mm; slice thickness = 0.5 mm; matrix size = 256 x 176; acquisition time = 5:31).

The same protocols, except the T2*WI based on MEDIC, were used for the *in vitro* MR visualization of SPIO-labeled cells injected into the phantoms.

### Quantitative analysis of intracerebral SPIO-labeled cell transplantation MRI data

MRI data analysis was performed using ImageJ software (Rasband, W.S., ImageJ, U. S. National Institutes of Health, Bethesda, Maryland, USA, http://imagej.nih.gov/ij/, 1997–2015). The area of signal hypointensity at the site of stereotaxic SPIO-labeled cell transplantation was measured using semi-automatic region selection. The volume V of the hypointensity zone was calculated by the summation of areas measured on adjacent cross-sections using the following formula: V = (S1+…+Sn) × (h+d), where S1,…,Sn–is area measured on slice n, h is the slice thickness, and d is the interval between the slices. The ratio of minimum to mean signal was measured using points close to the straight line, drawn parallel to the coil and perpendicular to the direction of stereotaxic injection as shown in S1 Fig. The minimum signal intensity was measured at the site of injection, and the mean signal intensity was calculated using values obtained from intact surrounding tissue.

### Histology

Immediately after MRI, animals were sacrificed by intraperitoneal injection of a lethal dose of Chloral hydrate and transcardially perfused with 0.1 M phosphate buffered saline (PBS, pH 7.4), followed by ice-cold 4% paraformaldehyde (PFA) in 0.1M PBS. The brains were removed, post-fixed at 4°C overnight in the same fixative, washed three times with PBS. Сoronal 40 μm-thick sections were cut the HM 650 v vibratome (Microm GmbH, Berlin, Germany). Every second section through the transplantation site was used for fluorescence microscopy, every third section—for fluorescent confocal microscopy, and each fourth section—for conventional bright-field microscopy. Prior to fluorescence microscopy, slide-mounted tissue sections were counterstained by incubating for 10 min at 20–22°C with DAPI solution (2 μg/mL, Sigma) to visualize cell nuclei. The fluorescence histological images were acquired with the BZ-9000E fluorescence microscope (Keyence, Japan). Fluorescence confocal micrographs were captured with the Nikon A1R MP+ laser scanning confocal microscope equipped with a 405, 488, 561, 638 lasers and Plan Apo20x/0.75 Dic N, Apo IR 60x/1.27 WI and Apo TIRF 60x/1.49 oil Dic objective lenses. To visualize the iron microspheres, Perls’ Prussian blue staining was performed. Tissue sections were rinsed with deionized water and immersed in Perls’ solution containing 2% potassium ferrocyanide and 2% HCl for 10 minutes. Sections were then rinsed with deionized water and counterstained with Neutral Red (Sigma) for 3 minutes. Bright-field images were acquired using Keyence BZ-9000E microscope.

### Statistical analysis

Statistical analysis of cell viability and proliferation data was performed by one-way ANOVA using GraphPad Prism software (GraphPad, San Diego, CA, USA).

Statistical analysis of MRI data was performed with Statistica 10 (StatSoft, USA) using Wilcoxon matched pairs test for dependent samples and Mann-Whitney U test for independent samples. For all the results statistical significance was set at p < 0.05.

## Results

### Labeling of hMSCs with SPIO microparticles and its influence on cell viability and proliferation

**Fig 1:**
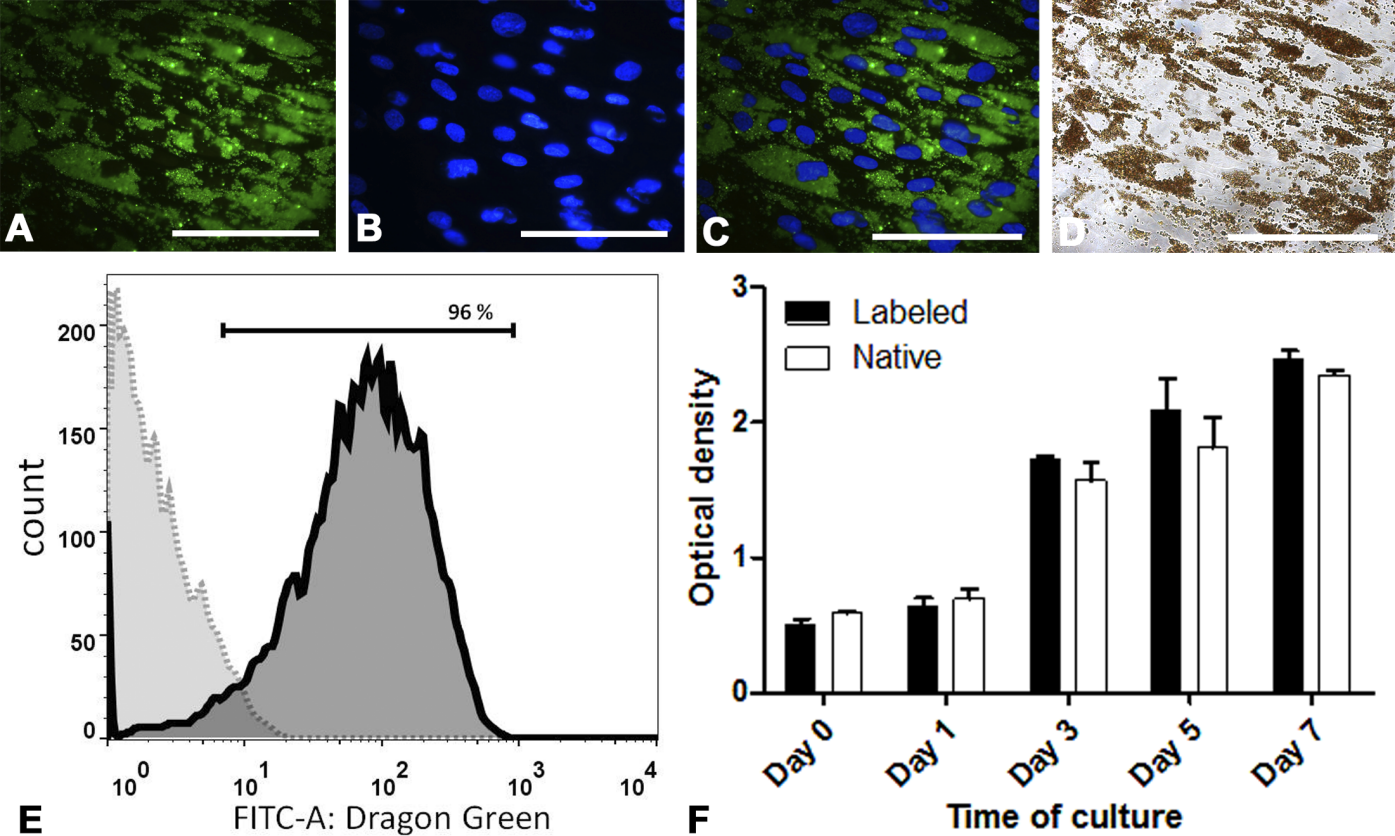
The efficacy of hMSCs labeling with MC03F microparticles and the effects of labeling on cell viability and proliferation. (A)-(C): fluorescent microscopy of a hMSC culture 24 hours after labeling with MC03F microparticles. (A) MC03F microparticles (Dragon Green fluorescence). (B) Cell nuclei (DAPI blue fluorescence). (C) A and B merged. (D) Transmitted light microscopy of unstained hMSC culture (the same area as in A-C) 24 hours after labeling with MC03F microparticles. SPIO microparticles are visualized in the cytoplasm as brown spots around clear nuclei. (E) Flow cytometry analysis of cells 24 hours after labeling. The solid line presents data for labeled hMSCs and the dotted line—for unlabeled, control hMSCs. X-axis shows fluorescence intensity and Y-axis—cell counts. The plot demonstrates that about 96% of the cells contained Dragon Green fluorescent microparticles. (F) Influence of the labeling with MC03F microparticles on hMSCs viability and proliferation. Optical density (Y axis) is proportional to lactate dehydrogenase (LDH) activity in the cells and, hence, to the number of living cells. The presented histograms show that the numbers of living cells were not significantly different in labeled and control cultures indicating the absence of negative effects associated with labeling on cell viability and proliferation. The scale bars on all microphotographs mark 100 μm.

As clearly seen on Fig 1A–1C, 24 hours after labeling the cytoplasm of virtually all cells of hMSC cultures contained vast amounts of fluorescent microspheres. DAPI-stained nuclei seem to contain no microparticles. SPIO microparticles could also be visualized in the cell cytoplasm with conventional transmitted light microscopy (Fig 1D). Since cell nuclei remained totally clear, Fig 1D leaves no doubts that SPIO microspheres concentrate within the cytoplasm. The efficacy of labeling accurately assessed with flow cytometry is shown on Fig 1E. As follows from flow cytometry data, no less than 96% of the cells emitted green fluorescence indicating the presence of MC03F microparticles in the cytoplasm.

To quantitatively evaluate the influence of labeling on cell viability and proliferation, we compared the dynamics of growth of intact and labeled hMSCs. The number of live cells on different days (1, 3, 5, 7) after labeling was evaluated using the CytoTox 96 LDH (lactate dehydrogenase) assay, in which the absorbance at 490 nm is proportional to total LDH activity and, hence, to the number of living cells. The results are presented in Fig 1F. Obviously, the proliferation rates of hMSCs containing microspheres and control cells are not significantly different suggesting the absence of negative effects associated with labeling on cell viability and proliferation. Earlier we have shown that labeled hMSCs retained the SPIO microparticles for at least four weeks [33]. Moreover, labeling did not noticeably affect the cells’ ability to undergo osteogenic, chondrogenic, adipogenic, and neurogenic differentiation *in vitro* [33,34].

Importantly, optimum incubation times and microparticles concentrations for labeling of MSCs with SPIO microspheres from Bangs Laboratories reported by other research groups are similar to ours [14,35].

### Iron content per cell and per injection

Average iron content per cell detected spectrophotometrically as described in the Materials and Methods section was 3.8±0.3 pg/cell. Therefore, the total amount of iron injected with 10^1^, 10^2^, 10^3^, 10^4^, and 10^5^ SPIO-labeled cells was 0.038 ng, 0.38 ng, 3.8 ng, 38 ng, and 380 ng, respectively.

### *In vitro* MRI of the phantoms

**Fig 2:**
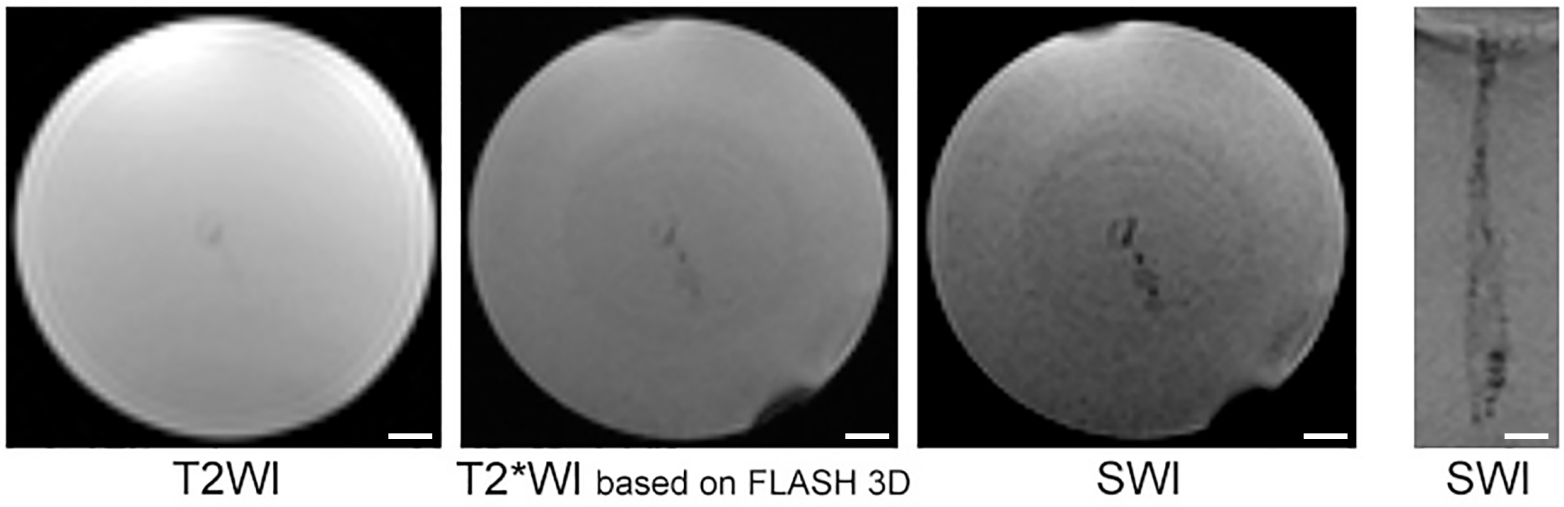
MR images of a phantom injected with 20 μl of saline containing 10^1^ hMSCs labeled with MC03F microspheres. The images were taken utilizing different MRI pulse sequences—T2WI, T2*WI based on FLASH 3D and SWI. Labeled hMSCs are visualized as hypointense areas in the isointense Spherogel milieu. Images captured at all three pulse sequences are presented in axial projection (three left panels). In addition, the coronal projection of the SWI is presented (right panel). The scale bars represent 1 mm.

Representative MR images of a phantom after injection of 10^1^ SPIO-labeled hMSCs obtained using T2WI, T2*WI based on FLASH 3D, and SWI are shown in Fig 2. Due to the ability of SPIO microspheres to create local disturbance of the magnetic field and to reduce T2* relaxation time, injected SPIO-labeled cells cause MR signal reduction and can be visualized at MR images as dark areas of hypointensity. Images obtained using all three pulse sequences are presented in axial projection. In addition, susceptibility weighted image is given in coronal projection. Obviously, SWI allowing easy detection of just several cells injected into a phantom, is the most sensitive of the tested pulse sequences.

### MRI of SPIO-labeled cells transplanted into the rat striatum

**Fig 3:**
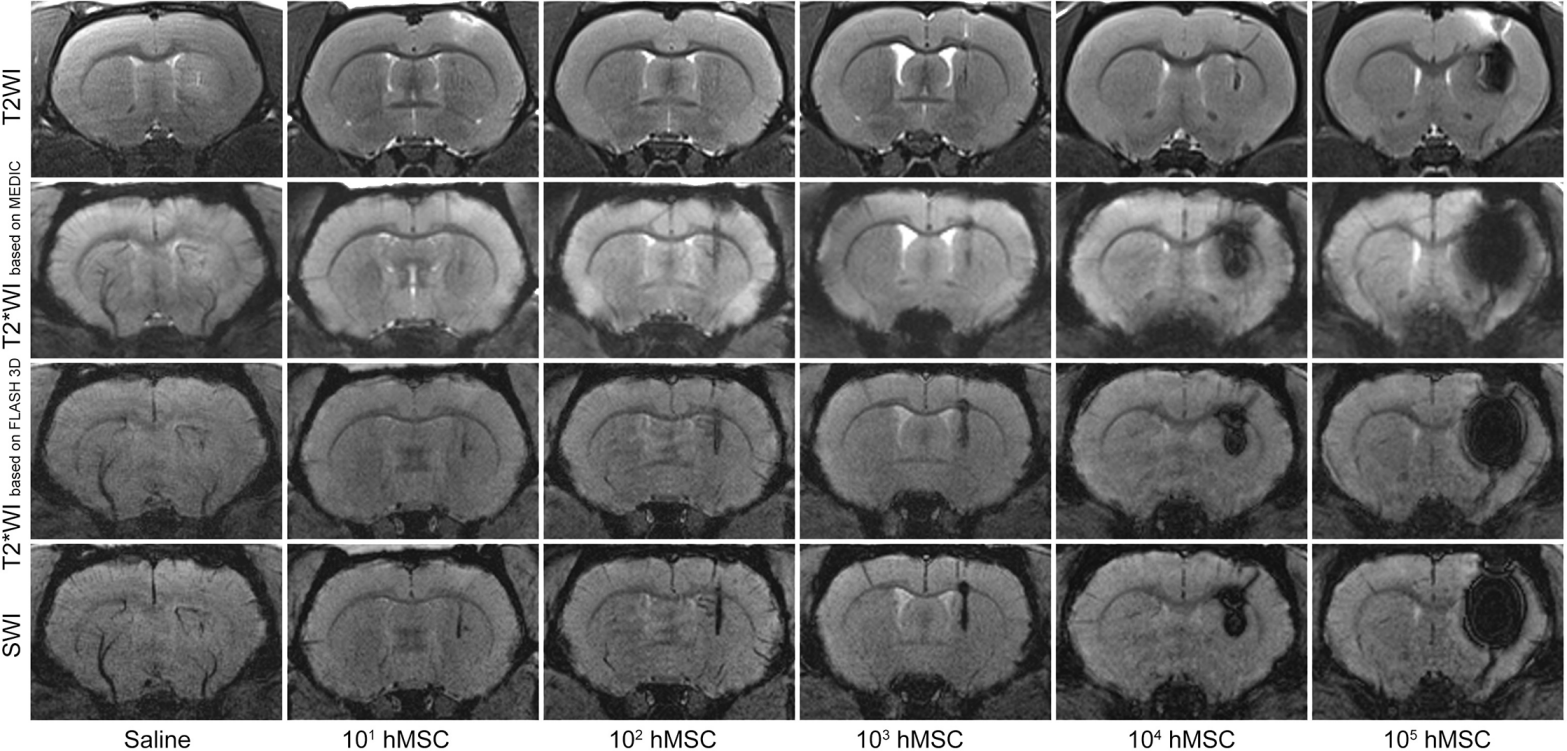
MR images of live rat brains after stereotaxic injection of 20 μl saline or different quantities (from 10^1^ to 10^5^) of SPIO-labeled hMSCs in 20 μl saline into the right striatum. The images were taken immediately after cell transplantation utilizing different MRI pulse sequences—T2WI, T2*WI based on MEDIC, T2*WI based on FLASH 3D, and SWI.

Fig 3 demonstrates characteristic MR images of normal live rat brain obtained immediately after stereotaxic injection of 20 μl saline or varying quantities of SPIO-labeled hMSCs (from 10^1^ to 10^5^ cells) in 20 μl saline into the right striatum. Images were taken using four different pulse sequences—T2WI, T2*WI based on MEDIC, T2*WI based on FLASH 3D and SWI. Similar to phantoms, the hypointensity areas at MR images of brains injected with labeled cells correspond to hMSCs transplantation sites. The size of the hypointensity regions correlates with the number of transplanted hMSCs and depends on the pulse sequence used. Large enough quantities of labeled hMSCs can create disturbance of the local magnetic field within a volume by far surpassing the volume occupied by the cells—a feature called «blooming effect». Small hypointensity areas on the SWI and T2*WI based on FLASH 3D images of the right striatum of saline-injected rat were probably produced by traumatic hemorrhages caused by the needle.

**Fig 4:**
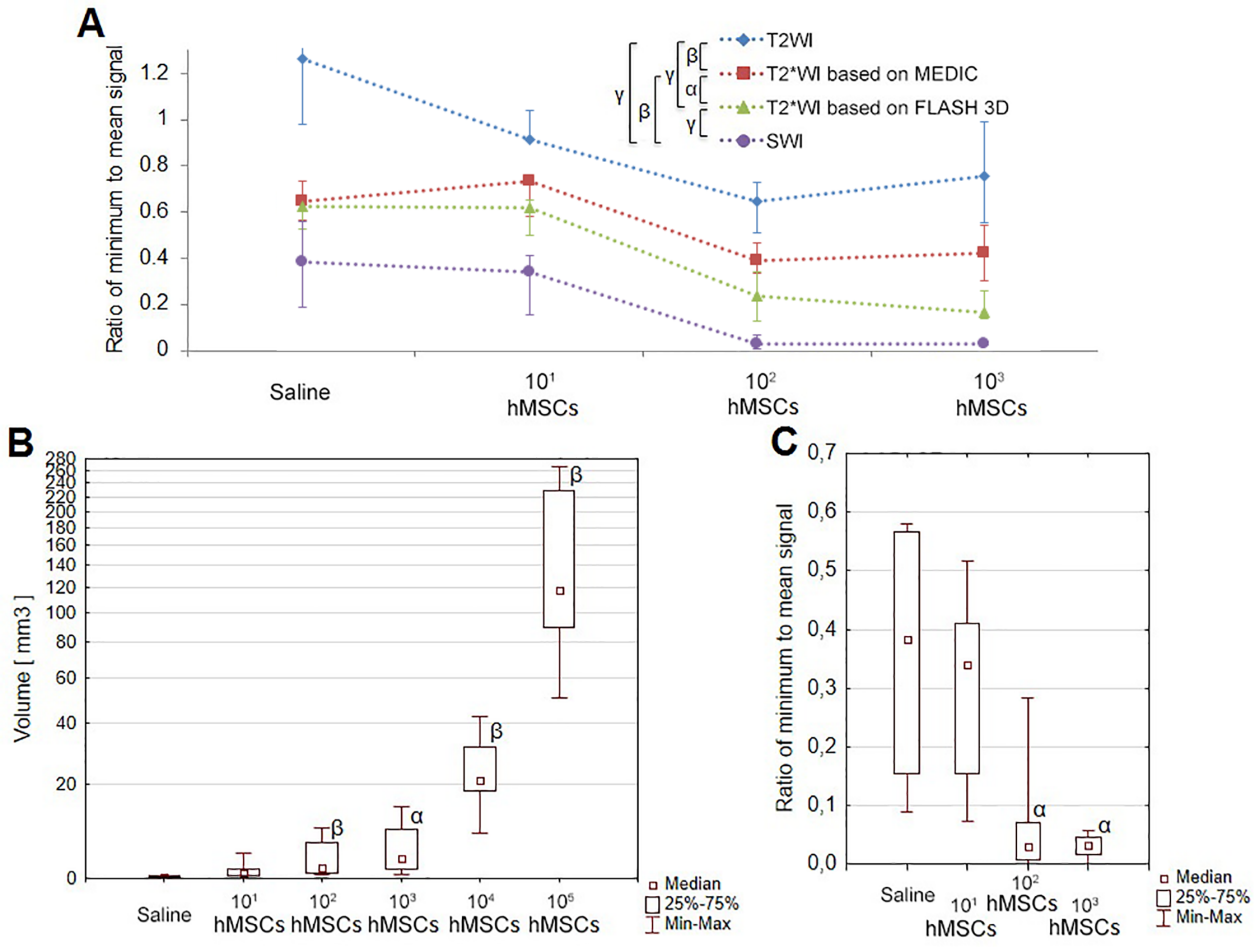
Quantitative evaluation of MRI data. (A) The medians of the values of the ratio of minimum to mean signal calculated from data obtained using different pulse sequences after injection of 10^1^, 10^2^ or 10^3^ of SPIO-labeled hMSCs or saline into the rat striatum. Data for cell concentrations higher than 10^3^ are not shown on the graph, because for them the minimum signal was zero for most MRI pulse sequences. Whiskers on the plot represent interquartile range. (B) Volumes of the hypointensity zones calculated from SWI data obtained after injection of varying quantities of SPIO-labeled hMSCs or saline into the rat striatum (box and whisker plot). (C) Values of the ratio of minimum to mean signal calculated from SWI data obtained after injection of 10^1^, 10^2^ or 10^3^ of SPIO-labeled hMSCs or saline into the rat striatum (box and whisker plot). Greek letters show statistical significance of differences between pulse sequences (A), or saline-injected and SPIO-labeled cell-transplanted rats (B, C): α –p<0.05; β –p<0.01; γ –p<0.001.

The sensitivities obtained using different pulse sequences were quantitatively estimated by measuring the ratio of the minimum signal intensity at the site of transplantation to mean signal intensity in the adjacent brain tissue (Fig 4A). Ratios of minimum to mean signal calculated from SWI data were significantly lower than those assessed using alternative pulse sequences (Fig 4A), demonstrating higher sensitivity of SWI in SPIO-labeled cells detection.

We also studied the ability of SWI to distinguish between signals produced by traumatic microhemorrhages inflicted by the syringe needle and different numbers of transplanted hMSCs. Comparison of the volumes of the hypointense zones and the values of the ratio of minimum to mean signal are shown in Fig 4B and Fig 4C. Data presented in Fig 4B and 4C indicate that after transplantation of 10^2^ or higher numbers of hMSCs hypointense areas captured using SWI were significantly different from those obtained from saline-injected rats. However, the signal from 10^1^ hMSCs could not be statistically distinguished from that from traumatic brain microhemorrhage inflicted by the syringe needle.

### Correlation between MRI and histology data in SPIO-labeled hMSCs brain injection experiments

**Fig 5:**
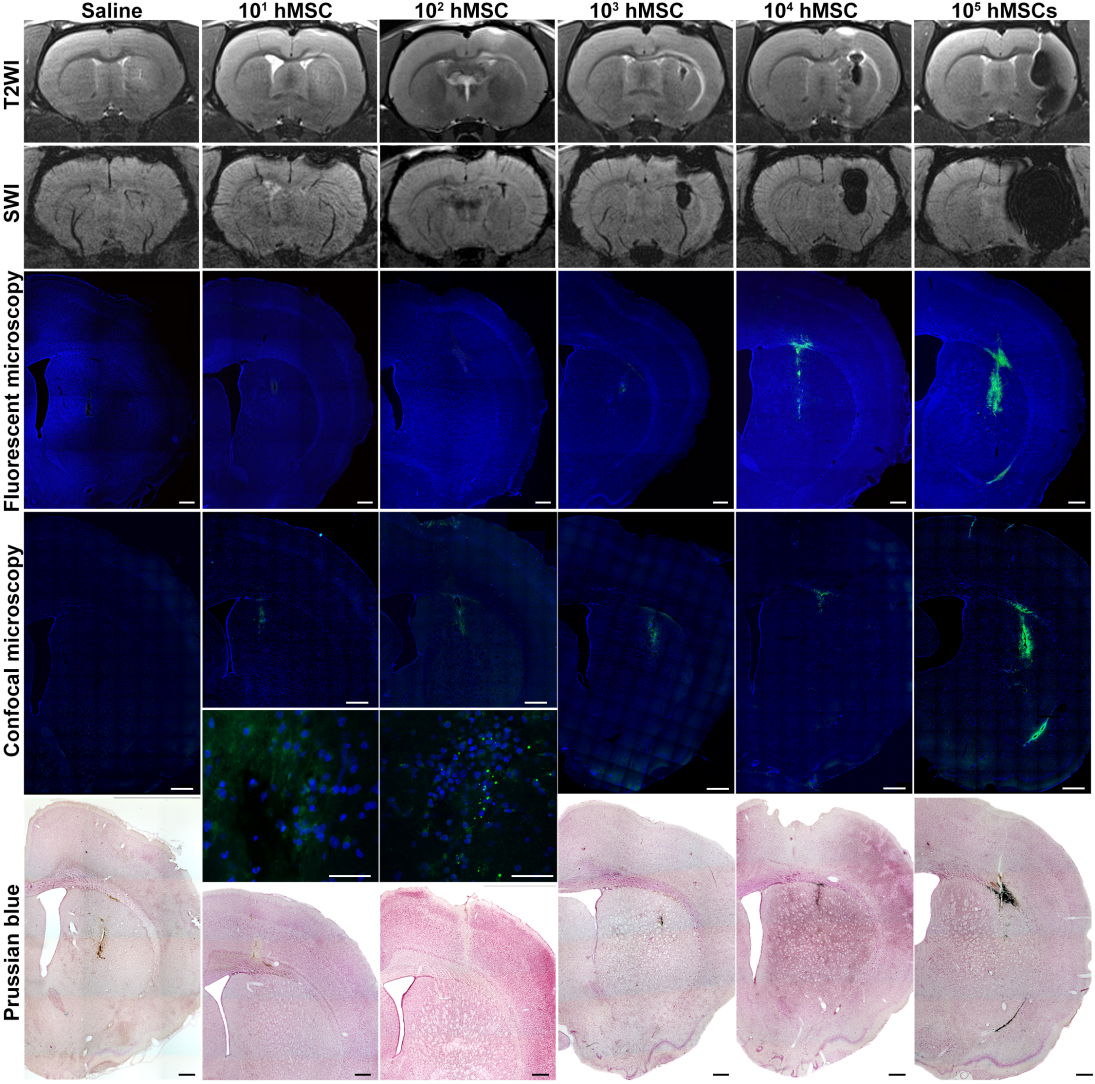
MR and histological images of the sites of stereotaxic injection of 20 μl of saline or varying numbers of SPIO-labeled hMSCs suspended in 20 μl of saline into the right striatum of rats. Top panel row: T2WI. Second from top panel row: SWI. Middle panel row: conventional fluorescence microscopy. Cell nuclei stained with DAPI look blue. Transplanted hMSCs containing MC03F microparticles in their cytoplasm emit green fluorescence. Second from bottom panel row: confocal fluorescence microscopy. Bottom panel row: conventional microscopy of Perls’ Prussian Blue stained sections. The scale bars represent 500 μm in panoramic views of coronal sections and 50 μm in high-magnification images.

Cells used in this study were labeled with microspheres consisting of a SPIO core covered with a shell containing the Dragon Green fluorescent dye. They can be visualized in different ways on histological sections. Three microscopic techniques, namely, conventional fluorescent microscopy, confocal fluorescent microscopy, and transmitted light microscopy of Perls’ Prussian blue stained sections were applied to expose transplanted hMSCs in rat brain tissue in order to verify MRI results. Fig 5 shows histological images of the sections across the sites of injection of varying numbers of labeled hMSCs (three lower panel rows) in comparison with corresponding MR images taken utilizing T2WI (upper panel row) and SWI (second from top panel row) pulse sequences. At the lower left panel iron is visible due to hemoglobin biodegradation inside a microhemorrhage. Injections made directly into the live brain tissue cause microhemorrhages of varying severity at the injection site. Perls’ Prussian blue stain is unable to distinguish between iron in hemorrhages and SPIO label. And because hemorrhages may vary in size, the site of saline injection sometimes looks even darker than the site of injection of a small number of SPIO labeled cells. Bigger cell numbers (10^3^−10^4^ on Fig 5) mask magnetic field disturbances caused by microhemorrhages. Accordingly, staining for iron content with Perls’ Prussian blue could reveal no less than 10^3^ labeled cells or about 3.8 ng of iron (lower panel row), and so did conventional fluorescent microscopy (middle panel row). If the number of cells was under 10^3^, microhemorrhages at the injection site could be mistaken for labeled cells, because both produced magnetic field disturbances of similar intensity. Confocal fluorescent microscopy allowed detection of 10^1^−10^2^ cells (second from bottom panel row). However, in some cases detection of 10^1^ cells by confocal fluorescent microscopy was somewhat obstructed by the interfering low intensity autofluorescence present after saline injection.

All three histological methods used showed the presence of labeled cells at the sites of hMSCs transplantation in the same locations as revealed by MRI. Taken together, histological data provided complete verification of MRI results. The sensitivity of the confocal fluorescent microscopy by far surpassed the detection limits of other histological techniques and was similar to that of SWI.

### MRI of SPIO-labeled cells after intra-arterial transplantation

**Fig 6:**
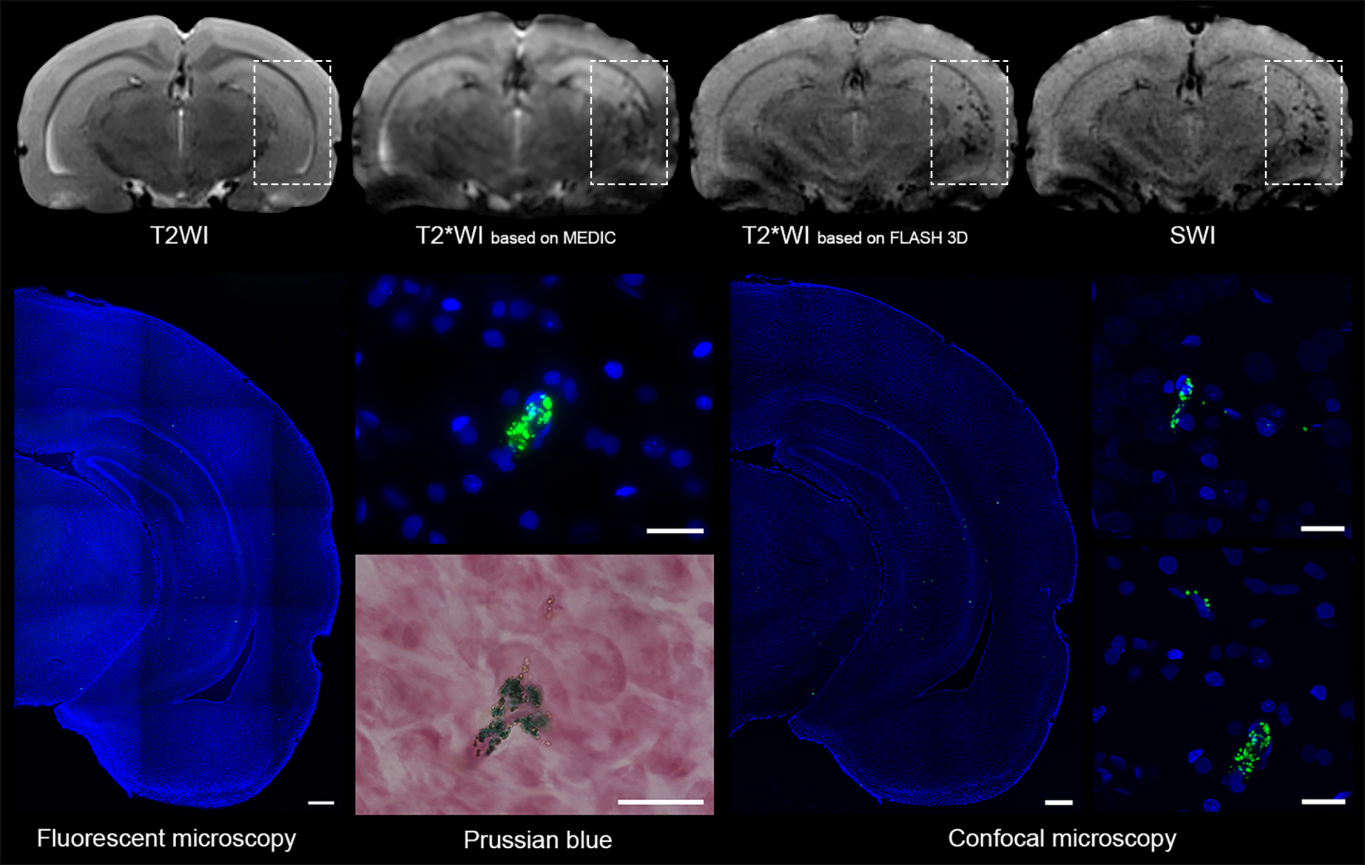
MR and histological images of a rat brain taken after intra-arterial administration of 5x10^5^ hMSCs labeled with MC03F microparticles via the right internal carotid artery. MRI was performed immediately after cell transplantation and euthanasia–immediately after MRI. Top panel row, left to right: T2WI, T2*WI based on MEDIC, T2*WI based on FLASH 3D, and SWI images. Rectangles indicate the area of labeled cells distribution. Bottom panel, left to right: conventional fluorescence microscopy (low and high magnification), bright-field microscopy of Perls’ Prussian Blue stained sections, confocal fluorescence microscopy (low and high magnification). The scale bars in panoramic views of coronal sections represent 500 μm and at high-magnification pictures they correspond to 50 μm.

Immediately after intra-arterial injection of 5x10^5^ hMSCs via the right internal carotid artery labeled cells could be visualized with applicable MRI techniques and were distributed mainly in the basal ganglia area and in the cerebral cortex of the right hemisphere (Fig 6, top row). SPIO-labeled hMSCs could be visualized as hypointense spots on images taken using T2*WI based on MEDIC, T2*WI based on FLASH 3D, and SWI. As evident from Fig 6, SWI and T2*WI based on FLASH 3D were most sensitive permitting detection of small groups of labeled cells, probably even single cells. T2WI, because of its low sensitivity, revealed no SPIO-labeled cells at all. MRI data were confirmed by the results of histological examination (Fig 6, bottom row). All three histological techniques used (conventional fluorescence microscopy, bright-field microscopy of Prussian Blue stained tissue sections, and confocal fluorescence microscopy) showed the presence of the SPIO label exactly at the same sites as found by MRI.

**Fig 7:**
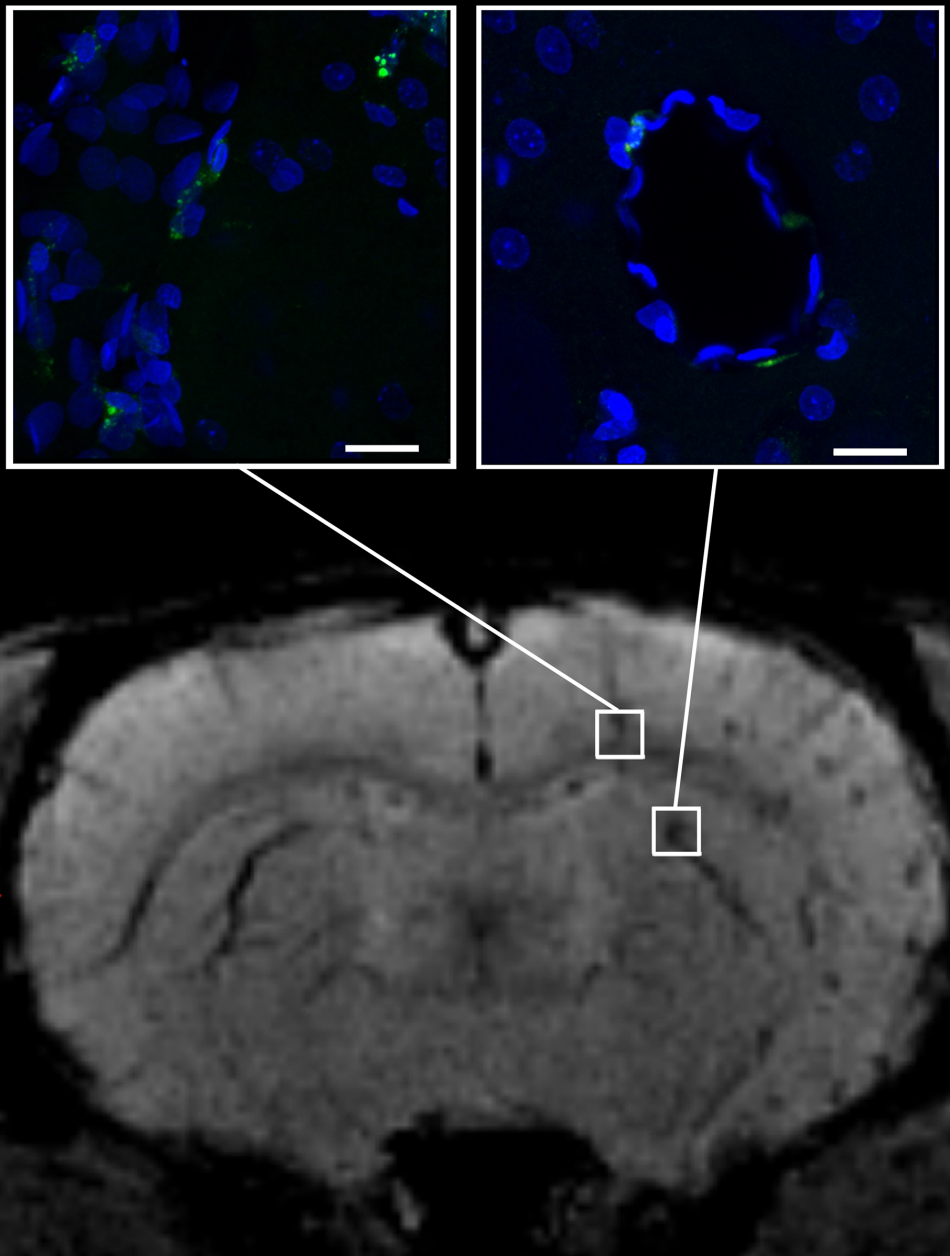
SWI and high-magnification confocal fluorescence microscopy of normal rat brain immediately after intra-arterial injection of 5x10^5^ hMSCs labeled with MC03F microparticles. SWI allows detection of small groups and even single hMSCs found in brain tissue close or around cerebral blood vessels. The scale bars represent 50 μm.

As seen at Fig 7, some labeled hMSCs were localized around or close to blood vessels, suggesting rapid migration of transplanted cells from circulation into the brain parenchyma through the intact blood-brain barrier. Fig 7 clearly demonstrates the possibility of MRI visualization of single SPIO-labeled transplanted cells in the recipient’s brain.

### MRI and histological visualization of hMSCs double-labeled with MC03F microparticles and PKH26

**Fig 8:**
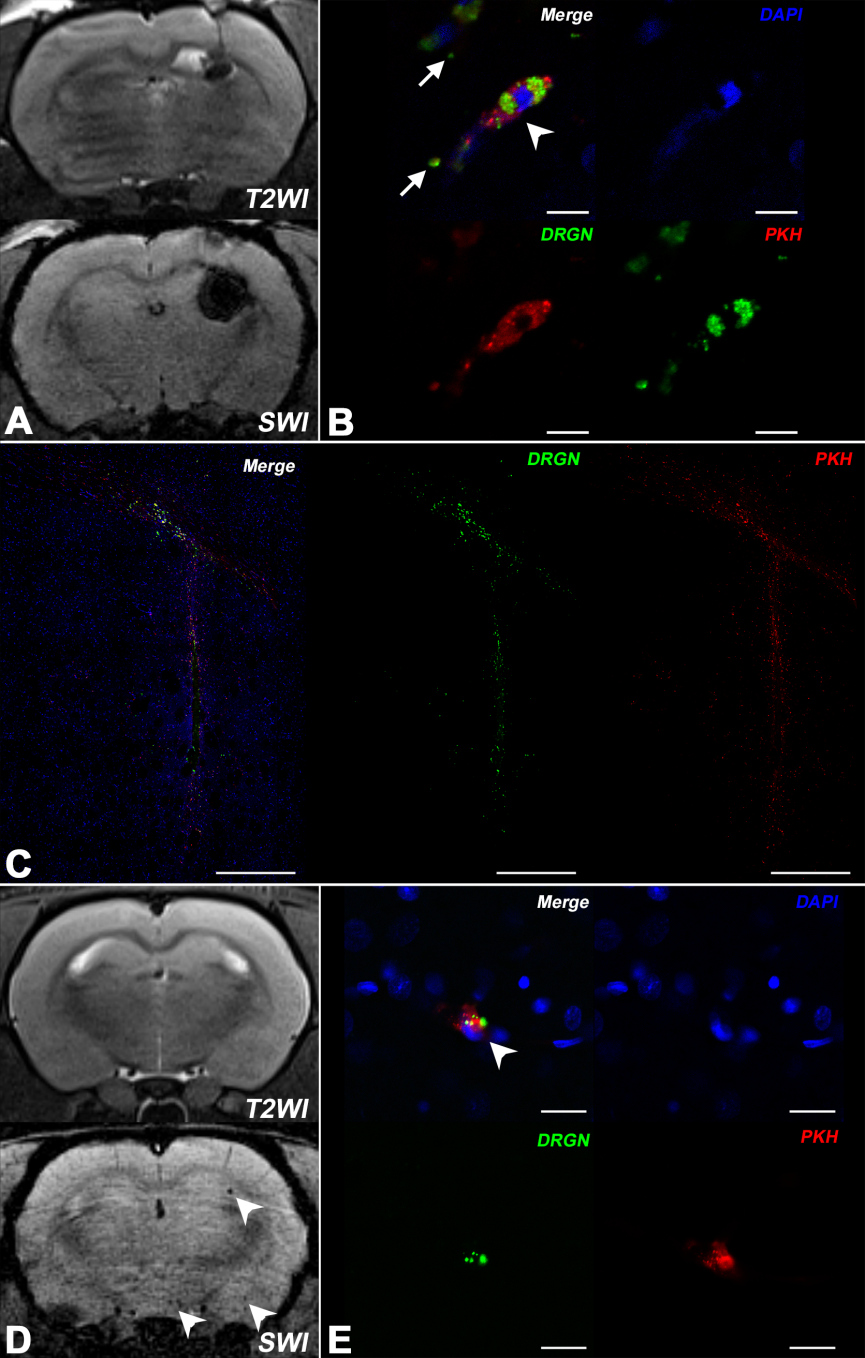
MR and confocal fluorescence microscopy images of rat brain after intracerebral and intra-arterial transplantation of hMSCs double-labeled with SPIO microparticles and PKH26. (A) MRI of rat brain (T2WI and SWI) immediately after intracerebral injection of 10^5^ hMSCs double-labeled with MC03F SPIO microparticles and PKH26. Hypointense regions indicate the location of SPIO labeled cells or probably extracellular SPIO microspheres. (B) High-magnification confocal micrographs of the hMSCs injection site. The same rat brain as in fig A. White arrowhead points to a double-labeled hMSC (membrane dye PKH26 is red, SPIO microparticles in the cytoplasm are green, and cell nucleus stained with DAPI is blue). Single clusters of extracellular iron can also be visualized (white arrows). The scale bars represent 10 μm. (C) Confocal panoramic micrographs of the hMSCs injection site (the same rat brain as in fig A). Labeled hMSCs are located along the track of the injection needle and in corpus callosum. The scale bars represent 500 μm. (D) MRI of rat brain (T2WI and SWI) after intra-arterial transplantation of 10^5^ hMSCs labeled with SPIO-microparticles and PKH26. The white arrows on the SWI picture indicate the location of SPIO labeled cells. (E) High-magnification confocal micrographs (the same rat brain as in fig D) of transplanted hMSCs. White arrowhead points at double-labeled cells (membrane dye PKH26 is red, SPIO microparticles in the cytoplasm are green, and cell nuclei stained with DAPI is blue). The scale bars represent 20 μm.

To confirm that SPIO microparticles remain localized inside hMSCs after transplantation, we performed additional labeling of cells with lipophilic membrane cell tracking dye PKH26. MR images of the injection site taken after intracerebral transplantation of 3x10^5^ double-labeled cells transplantation show the “blooming effect” (Fig 8A). As seen at the high resolution confocal fluorescent microscopy images (Fig 8B), SPIO-labeled cells (white arrowhead) carry two fluorescent labels: the Dragon Green label associated with SPIO particles inside the cytoplasm and the red membrane tracker PKH26. Co-localization of the two labels demonstrates that after intracerebral transplantation at least part of the SPIO label remains associated with hMSCs. However, small clusters of extracellular green fluorescent SPIO microspheres are also found (white arrows at Fig 8B). Extracellular SPIO can be mistaken for SPIO-labeled hMSCs on MR images. Panoramic confocal fluorescent microscopy images of the injection site (Fig 8C) demonstrate close co-localization of the two fluorescent labels. After intra-arterial transplantation double-labeled MSCs can be observed *in vivo* with SWI, while T2WI is not sensitive enough to visualize single hMSCs or small groups of transplanted cells (Fig 8D). It should be noted, that T2*WI based on FLASH 3D can also provide single cells detection, but the quality of images is poorer compared to SWI. The comparison of T2*WI based on FLASH 3D and SWI images is presented at S2 Fig in Supporting information). High resolution confocal microscopy images (Fig 8E) show double-labeled hMSCs (white arrowhead), but no extra-cellular SPIO microparticles.

## Discussion

Our *in vitro* experiments with phantoms injected with SPIO-labeled hMSCs demonstrated higher sensitivity of SWI compared to methods utilizing other pulse sequences, such as T2WI and T2*WI. SWI provided labeled cells detection threshold as low as 10^1^ cells at least. These findings are in accordance with the previously published studies demonstrating *in vitro* sighting of 10^1^ cells or even single cells [14,24–26]. Table 1 summarizes the results of published phantom studies concerning detection thresholds for SPIO-labeled cells. The minimum MRI-detectable number of SPIO-labeled cells injected into a phantom in a small volume depends on the type of MR scanner (clinical or experimental, magnetic field strength), the pulse sequence used for imaging (T2WI, T2*WI or SWI) [22,36], the type of the contrasting agent, and its amount per cell. In our study the average iron content per cell was 3.8±0.3 pg and the amount of iron detected in phantom experiments was 0.038 ng. Currently, a wide range of SPIO particles with varying sizes (from several nanometers up to micrometers), iron content, magnetic cores and coating is available commercially [37]. Different types of SPIO-containing particles are internalized by cells in varying quantities [15,36]. Most commonly used SPIO particles can deliver 2–9 pg Fe/cell [13,37,38], though higher uptake of iron up to 69.1 pg per 1 human MSC (citrate-coated SPIO nanoparticles, MagneticFluids, Germany) was reported [39], suggesting the likelihood of further MRI sensitivity improvement. Literature data summarized in Table 1 show, that the most sensitive detection of SPIO-labeled cells in phantom studies can be achieved using high field experimental MR system and SWI [19,21,22] and the results of our phantom experiments are in line with these findings.

**Table 1:** MRI detection thresholds of SPIO-labeled stem cells in phantom studies.

Reference	Type of cells and labels used	MR system type and pulse sequence	Minimum number of cells detected and cells/μl detected
This study	hMSCs from human placenta—SPIO microspheres (MC03F, Bangs Laboratories, USA)	Experimental 7T - SWI	1x10^1^ / 0.5 in 1 μl
Hinds et al.[14]	Bone marrow MSCs—SPIO microspheres (0.9 μm, Bangs Laboratories, USA)	Experimental 11.7T - T2*	Single cell
Ariza de Schellenberger et al.[13]	Murine MSCs—Two types of hanmade SPIO and Resovist® (Resovist®, Bayer Healthcare, Germany)	Experimental 7T - T2* / Experimental 7T with CryoProbe™ - SWI	Single cell
Lobsien et al.[40]	Ovine bone marrow MSCs—Very small SPIO particles (VSOP) 5-nm (Ferropharm, Berlin, Germany)	Clinical 3T - T2* and SWI	5x10^2^ / 5 in 1 μl
Park et al.[22]	hMSCs from bone marrow—Feridex® SPIO nanoparticles (Bayer Healthcare Pharmaceuticals, USA)	Clinical 3T - SWI	6.5x10^2^ / 13 in 1 μl
Stroh et al.[15]	Mouse embryonic stem cells—Very small SPIO particles (VSOP) C200 (Ferro Pharma, Germany)	Experimental 17.6T - T2*	1x10^2^ / 50 in 1 μl
Argibay et al.[41]	Rat MSCs—Handmade coated magnetic nanoparticles (8.6±1.4 nm mean diameter)	Experimental 9.4T - T2*	1x10^5^ / 5 000 in 1 μl
Umashankar et al.[42]	Rat neural stem cell—MIRB (USPIO-based contrast reagent BioPAL Inc., USA)	Experimental 7T - T2*	1x10^5^ / 5 000 in 1 μl
(Magnitsky et al.[36]	Mouse neural stem cells—Feridex® SPIO nanoparticles (Bayer Healthcare Pharmaceuticals, USA)	Experimental 9.4T - T2*	1x10^2^ / -
Kallur et al.[43]	Human neural stem cells—Endorem® SPIO nanoparticles (Guerbet GmbH, Germany)	Experimental 11.7T - T2*	5x10^2^ / -
Magnitsky et al.[36]	Mouse neural stem cells—Feridex® SPIO nanoparticles (Bayer Healthcare Pharmaceuticals, USA)	Experimental 4.7T - T2*	5x10^2^ / -
Ha et al.[21]	hMSCs from bone marrow—Feridex® SPIO nanoparticles (Bayer Healthcare Pharmaceuticals, USA)	Clinical 3T - SWI	1.6x10^4^ / -
Byun et al.[19]	hMSCs from bone marrow—Feridex® SPIO nanoparticles (Bayer Healthcare Pharmaceuticals, USA)	Clinical 3T - SWI	6.3x10^4^ / -

This study included stereotaxic transplantation of 10^1^ to 10^5^ of the SPIO-labeled hMSCs in 20 μl saline or 20 μl of saline into the intact rat striatum and immediate MR examination of the brain utilizing several pulse sequences—T2WI, T2*WI based on MEDIC, T2*WI based on FLASH 3D, and SWI. SWI proved to be the most sensitive method for the detection of stereotaxically delivered SPIO-labeled hMSCs among all methods tested. Interestingly, in the case of intracerebral administration of large quantities of SPIO-labeled cells higher sensitivity of SWI can be a disadvantage. Comparison of histology and MRI data shows that the size of the hypointense zones visualized at SWI images after transplantation of high numbers of labeled hMSCs can significantly exceed the real volume occupied by the injected cells accurately revealed by histological examination of tissue sections. This feature is known as the “blooming effect” [44], characterized by nonlinear increase of the hypointense area with increasing iron content. Thus, if SPIO-labeled cells are concentrated in one location, T2*WI or even T2WI can be a better choice for detection of cell transplant due to weaker “blooming effect”. Importantly, the dependence of the intensity of generated MR signal from local SPIO label concentration is non-linear even at low label concentrations [45], preventing direct use of MRI for quantification of local iron content in live tissues including brain.

Unlike the case in phantom experiments, *in vivo* MR visualization of SPIO-labeled hMSCs could be obscured by microhemorrhages or dilated veins. In our hands SWI provided detectable hypointense trace after intracerebral injection of as few as 10^1^ cells, but statistically validated threshold for the visualization of transplanted hMSCs was higher and constituted 10^2^ cells. This was caused by traumatic microhemorrhages at the injection site altering the MR signal and obscuring the signal from 10^1^ SPIO-labeled cells. In addition, it is difficult to guarantee that exactly 1, 2, 3 or 5 cells are injected into the brain tissue. Failure to differentiate small numbers of SPIO-labeled cells from hemorrhages is a serious limitation of cell tracking with MRI after intracerebral implantation of cells, especially in studies employing animal disease models associated with bleeding or hemorrhagic transformation of the ischemic zones, i.e. cerebral trauma or stroke models. However, single cell detection *in vivo* can be accomplished when there is no artificial brain trauma, as demonstrated in this study in the experiments with intra-arterial injection of labeled hMSCs via internal carotid artery. Like hemorrhages, cerebral veins also can be confused with SPIO-labeled MSCs on MR images. However, blood vessels are elongated structures and can be easily distinguished from labeled cells by analyzing the series of adjacent slices. Our data concerning SPIO-labeled cell tracking after systemic administration are largely in agreement with the recently published paper of Ariza de Schellenberger et al. [13], who also managed to track single MSCs in live brain tissue.

Table 2 summarizes available data concerning the sensitivity of MR detection of SPIO-labeled cells in live animal brain. Data listed in Table 2 demonstrate reduction of the SPIO-labeled cells visualization threshold with the growth of the magnetic field strength. Increase in the magnetic field strength boosts the magnetic susceptibility and augments the sensitivity of MRI T2*WI and SWI pulse sequences [16]. In our experiments we demonstrated high SPIO-labeled cell detection sensitivity in live brain using ultra high field MRI system and SWI. At lower magnetic field strength detection sensitivity is likely to decrease, but SWI would probably allow to maintain reasonably high sensitivity even with weaker field MRI systems, as it was shown in phantom experiments by Lobsein et al. [40].

**Table 2:** MR detection thresholds of SPIO-labeled stem cells in live animal brain.

Reference	Type of cells, labels used and cells delivery method	MR system type and pulse sequence	Minimum number of cells detected and cells/μl detected
This study	hMSCs from placenta—SPIO microspheres (MC03F, Bangs Laboratories, USA)—Intracerebral	Experimental 7T - SWI	1x10^2^ / 5 in 1 μl
This study	hMSCs from placenta—SPIO microspheres (MC03F, Bangs Laboratories, USA)—Intra-arterial	Experimental 7T - SWI	Single cell
Ariza de Schellenberger et al.[13]	Murine MSCs—Two types of hanmade SPIO and Resovist® (Resovist®, Bayer Healthcare, Germany)—Intra-arterial	Experimental 7T with CryoProbe™ - SWI	Single cell
Stroh et al.[15]	Mouse embryonic stem cells—Very small SPIO particles (VSOP) C200 (Ferro Pharma, Germany)—Intra-arterial	Experimental 17.6T - T2*	1x10^2^ / 50 in 1 μl
Park et al.[22]	hMSCs from bone marrow—Feridex® SPIO nanoparticles (Bayer Healthcare Pharmaceuticals, USA)—Intracerebral	Clinical 3T - SWI	2x10^2^ / 13 in 1 μl
Jendelová et al.[46]	Rat MSCs from bone marrow—Endorem® SPIO nanoparticles (Guerbet GmbH, Germany)—Intracerebral	Experimental 4.7T - T2*	- / 625 in 1 μl
Yin et al.[47]	Human adipose-derived stem cells—Ferumoxytol/Feraheme SPIO (AMAG Pharmaceuticals, USA)—Intracerebral	Clinical 3T - SWI (SWAN)	1x10^4^ / 2 000 in 1 μl
Hu et al.[12]	hMSCs from umbilical cord—handmade SPIO nanoparticles (80nm mean diameter)—In to the spinal cord	Experimental 7T - T2	1x10^5^ / 4 000 in 1 μl
Argibay et al.[41]	Rat MSCs—handmade coated magnetic nanoparticles (8.6±1.4 nm mean diameter)—Intracerebral	Experimental 9.4T - T2*	5x10^4^ / 5 000 in μl
Umashankar et al.[42]	Rat neural stem cell—MIRB (USPIO-based contrast reagent BioPAL Inc., USA)—Intracerebral	Experimental 7T - T2*	5x10^4^ / 50 000 in 1 μl
Cheng et al.[20]	hMSCs from bone marrow—Resovist® SPIO nanoparticles (Bayer Healthcare Pharmaceuticals, USA)—Intracerebral	Clinical 3T - SWI	5x10^7^ / 10 000 000 in 1 μl
Magnitsky et al.[36]	Mouse neural stem cells—Feridex® SPIO nanoparticles (Bayer Healthcare Pharmaceuticals, USA)—Intracerebral	Experimental 4.7T - T2*	5x10^2^ /
Zhu et al.[48]	Rat neural stem cells—Endorem® SPIO nanoparticles (Guerbet GmbH, Germany)—Intracerebral	Experimental 4.7T - T2*	1x10^6^ /

Although in culture hMSCs retained the SPIO microparticles for at least four weeks, histological data concerning intra-cerebral administration of double-labeled cells (Fig 8B) show that during transplantation some cells lose the SPIO label. Clusters of SPIO microparticles deposited at the injection site or close to it may be misinterpreted as transplanted cells at MR images. We did not encounter that problem after intra-arterial hMSCs transplantation. Histological examination of brain tissue showed that the membrane tracker PKH26 (red fluorescence) was always co-localized with SPIO microparticles (Dragon Green green fluorescence) and the cell nuclei tracer DAPI (blue fluorescence) showing that at least shortly after having crossed the blood-brain barrier transplanted hMSCs did not lose the SPIO label or disintegrate.

In our study, the thresholds of visualization of SPIO-labeled cells with SWI and confocal fluorescent microscopy were very close. The precision of these methods exceeded that of conventional fluorescent microscopy and conventional bright-field microscopy of the Prussian blue stained sections. It is noteworthy, that Perls’ Prussian blue stain commonly used to detect the presence of the SPIO label, is similar to MRI in its inability to distinguish signals from iron in hemorrhages and SPIO label [44,49,50].

Intra-arterial injection of cells via the internal carotid artery is an effective route of MSCs delivery to brain in preclinical studies of the effects of cell therapy in experimental brain disorders [51,52]. Due to optimization of the injection parameters completed in our preliminary experiments [32], we did not observe cerebral embolic events after cell administration. The vast majority of transplanted hMSCs was carried away from brain with blood flow, while a small fraction penetrated the brain tissue and soon after injection could be visualized using MRI. Histological analysis showed that MRI in its SWI modification allowed visualization of single cells and small groups consisting of several cells. As anticipated, T2WI failed to reveal any SPIO-labeled cells after intra-arterial administration of SPIO-labeled cell suspension because of its low sensitivity and diffuse distribution of transplanted hMSCs in the brain. Unlike intra-cerebral injection, intra-arterial administration did not cause traumatic microhemorrhages which could be confused with MR signal from labeled cells. Consequently, all hypointense spots on SWI with the exception of those produced by cerebral veins, should correspond to the localization of the SPIO label. The veins can be relatively easily identified by their size and localization. Histological examination carried out immediately after intra-arterial administration of labeled hMSCs and MRI showed that immediately after transplantation hMSCs were localized in the proximity of small blood vessels. The ability of MSCs to cross the intact blood-brain barrier is in accordance with other studies [53].

We have shown that hMSCs retained the SPIO microparticles for at least four weeks *in vitro*. But *in vivo* SPIO-labeled hMSCs can be destroyed by the immune system. SPIO-labeled cells or extracellular SPIO label can be taken up by local macrophages. In the present study we did not assess that issue, because the goal was to find the best way of visualization of intact SPIO-labeled transplanted cells and we tried to minimize the time interval between hMSCs transplantation and MRI or tissue fixation in order to limit the alterations that might occur to the labeled cells. According to the available literature data, the uptake of SPIO labeled cells by the cells of the host immune system is minor (around the 10% of the cells) [54–57]. Nevertheless, this problem should be taken into account in more prolonged cell migration and homing studies.

In summary, this study involved the quantitative and qualitative comparison of the applicability of several iron sensitive MRI techniques for the *in vivo* visualization of transplanted SPIO-labeled cells in rat brain. We have shown that SWI, due to its high sensitivity and resolution, presents the most accurate way of MR visualization of SPIO-labeled cells after intracerebral and intra-arterial administration. In intracerebral administration studies, the visualization threshold was 10^2^ cells due to some limitations of this method, such as the “blooming effect”, inability to differentiate SPIO label from cerebral microbleeds or extra-cellular tissue iron deposits, and difficulties with determination of exact cell numbers at very low cell concentrations. SWI also presents a method of detection of small groups of labeled cells and single cells after intra-arterial administration. Thus, despite some restrictions, SWI can be a method convenient for monitoring migration and homing of transplanted SPIO-labeled MSCs within the brain. However, in this study we concentrated on the evaluation of the applicability of different MRI techniques to the *in vivo* detection of SPIO-labeled transplanted MSCs and, therefore, we performed MRI as soon as possible after cell transplantation in order to prevent any potential translocation of SPIO label due to excretion or capture by host cells, such as macrophages. To verify MRI data with histological analysis we performed euthanasia and brain tissue fixation immediately after MRI. Studies of SPIO-labeled cell migration and homing at longer post-transplantation time intervals are ongoing.

## Supporting information

S1 Fig Calculation of the ratio of minimum to mean signal. (A) Axial SWI image of rat brain at the site of stereotaxic injection of SPIO-labeled hMSCs. Signal intensity measurements were performed using ImageJ along the yellow line drawn parallel to the coil and perpendicular to the direction of stereotaxic injection. (B) Plot representing the variation of MR signal intensity along the yellow line shown in A. Minimum signal intensity was measured at the site of injection; mean signal intensity in intact surrounding tissue was calculated using values obtained at the distance of about two pixels right and two pixels left from the peak (red points).
(TIF)

S2 Fig MR images of rat brain after intra-arterial transplantation of hMSCs labeled with SPIO microparticles. Comparison of the ability of the three different pulse sequences to detect single labeled cells or small groups of labeled cells. White arrows indicate the location of SPIO labeled cells. Both T2*WI based on FLASH 3D and SWI allow detection of single labeled cells or small groups of labeled cells, but their visibility is higher with SWI. SWI detects more single cells or small cell clusters than T2*WI based on FLASH 3D.
(TIF)
