# Supplementary figures and images for: Methodological aspects of MRI of transplanted superparamagnetic iron oxide-labeled mesenchymal stem cells in live rat brain
Source: PLoS One. 2017 Oct 19;12(10):e0186717. doi: 10.1371/journal.pone.0186717 (PMC5648235; doi:10.1371/journal.pone.0186717)

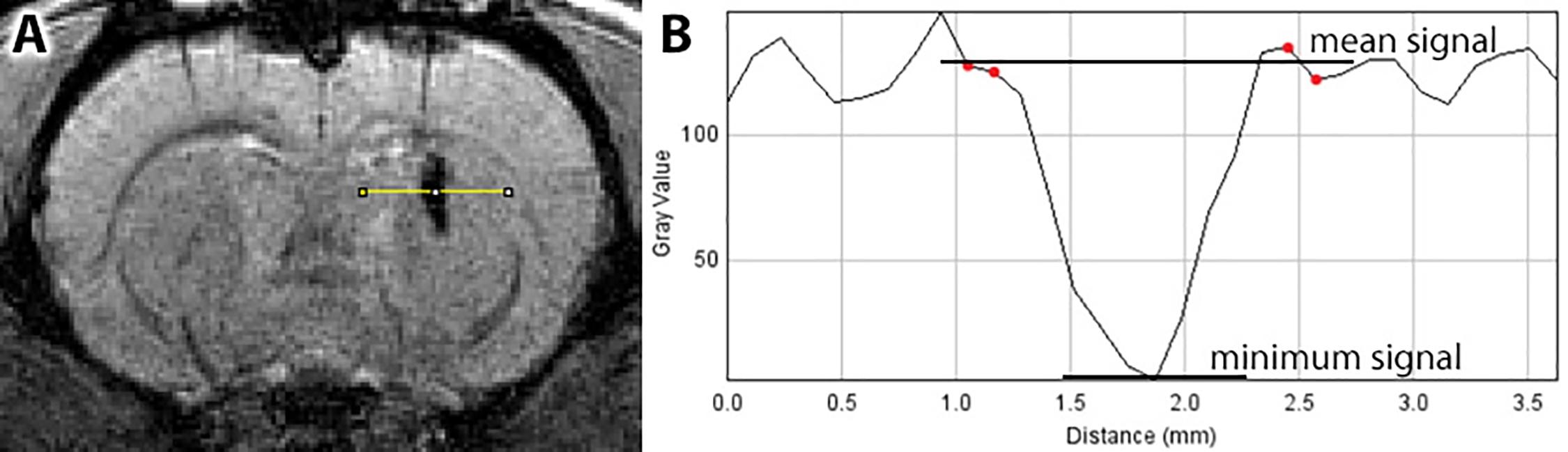

Supplement: S1 Fig — (A) Axial SWI image of rat brain at the site of stereotaxic injection of SPIO-labeled hMSCs. Signal intensity measurements were performed using ImageJ along the yellow line drawn parallel to the coil and perpendicular to the direction of stereotaxic injection. (B) Plot representing the variation of MR signal intensity along the yellow line shown in A. Minimum signal intensity was measured at the site of injection; mean signal intensity in intact surrounding tissue was calculated using values obtained at the distance of about two pixels right and two pixels left from the peak (red points). (TIF) [file pone.0186717.s001.tif]

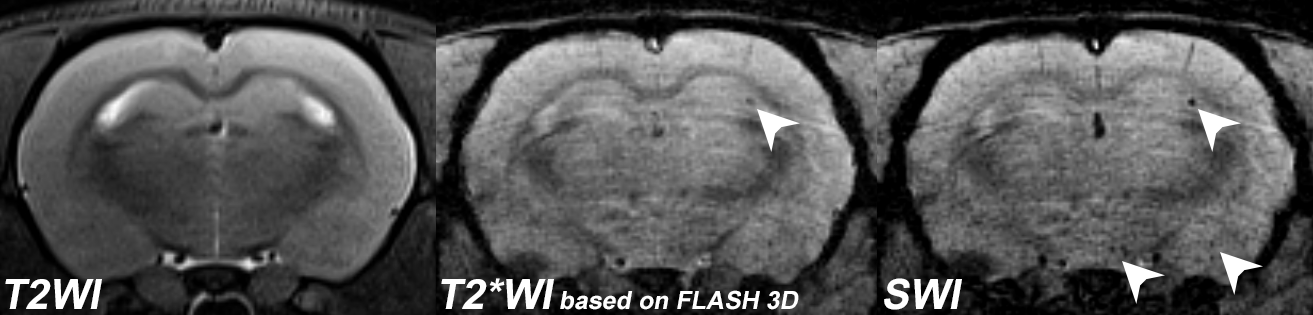

Supplement: S2 Fig — Comparison of the ability of the three different pulse sequences to detect single labeled cells or small groups of labeled cells. White arrows indicate the location of SPIO labeled cells. Both T2*WI based on FLASH 3D and SWI allow detection of single labeled cells or small groups of labeled cells, but their visibility is higher with SWI. SWI detects more single cells or small cell clusters than T2*WI based on FLASH 3D. (TIF) [file pone.0186717.s002.tif]
